# Dynamic Self-Assembly and Stimuli-Responsive Disassembly of Bioactive-Loaded Cubosomes in Biomimetic Media Traced by Real-Time Small-Angle X‑ray Scattering and Cryogenic Transmission Electron Microscopy

**DOI:** 10.1021/acsami.5c18735

**Published:** 2025-12-12

**Authors:** Rafael R.M. Madrid, Angelina Angelova, Borislav Angelov, Gouranga Manna, Patrick D. Mathews, Omar Mertins

**Affiliations:** a Laboratory of NanoBioMaterials - LNBM, Department of Biophysics, Paulista Medical School, 58804 Federal University of Sao Paulo, Sao Paulo 04023-062, Brazil; b Institut Galien Paris-Saclay, CNRS, 27051 Université Paris-Saclay, Orsay 91400, France; c Department of Structural Dynamics, Extreme Light Infrastructure ERIC, Dolni Brezany 18221, Czech Republic; d ESRF, The European Synchrotron, 71 Avenue des Martyrs, Grenoble 38000, France; e Institute of Bioscience, 28105 Sao Paulo State University, Botucatu 18618-689, Brazil

**Keywords:** self-assembly/disassembly, stimuli-responsive liquid crystalline structures, dynamic gastro-intestinal environment, structural fate of advanced materials in simulated biological environments, structural transitions, *Aloe vera* polysaccharides, pH-responsive coating of lipid nanoparticles

## Abstract

Designing advanced functional materials capable of passing through complex biological environments requires a deep understanding of their dynamic structural behavior *in situ*. We investigate pH-responsive core–shell cubosomes for oral drug delivery applications. These nanoparticles comprise a lipid-based core of cubic *Im3m* liquid crystalline structure and are coated with a chitosan-*N*-arginine/alginate polyelectrolyte shell (PS). The cubosomes encapsulate varying concentrations (0–30% w/w) of *Aloe vera*-derived acemannan, an immunomodulatory macromolecular drug. Utilizing synchrotron small-angle X-ray scattering and cryogenic transmission electron microscopy, we performed an advanced spatiotemporal analysis, which focused on their nanoscale structural evolution under simulated gastric (pH 2.5) and intestinal (pH 7.4) conditions. The interactions with key individual gastrointestinal components, including mucins, pepsin, bile salts, and pancreatin, were systematically examined. Our results demonstrate that acemannan incorporation and environmental pH significantly modulate cubosome structure and heterogeneity (phase coexistence) during disassembly. The pH-responsive polyelectrolyte shell imparts notable structural stability against pepsin and mucins at pH 2.5, ensuring functional gastric protection. However, under intestinal conditions (pH 7.4), bile salt-mediated solubilization caused complete disassembly. Pancreatic lipase-induced digestion triggered a remarkable time-dependent phase transition from a cubic (*Im3m*) to an inverted hexagonal (H_II_) topology in PS-cubosomes containing 30% acemannan. A simulated duodenum mixture induced lamellar phases at pH 2.5 for acemannan-loaded systems but led to complete disassembly at pH 7.4, primarily driven by bile salts. Deconvoluting these structural responses over time provides crucial insights into their mechanistic nature. It clarifies pH-dependent stability and component-specific disassembly pathways. The achieved understanding is crucial for designing advanced stimuli-responsive lipid/biopolymer nanomaterials that facilitate efficient oral delivery.

## Graphical abstract

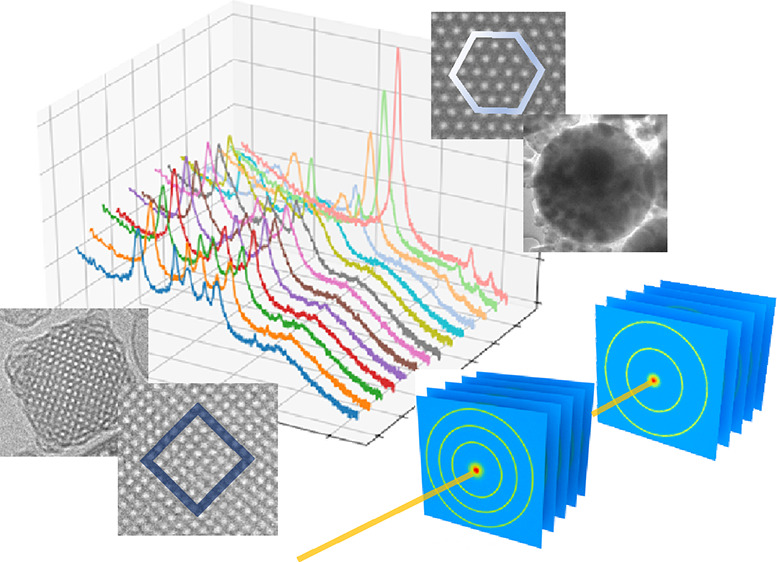

## 1 Introduction

Stimuli-responsive self-assembled biomaterials offer powerful platforms for controlling the delivery of therapeutic agents within complex biological environments.^1–6^ Among them, lipid-based nanoparticles (LNPs) with internally nanostructured cores and sensitive shells stand as a significant breakthrough in nanoarchitectures for the delivery of bioactives.^7–10^ pH-responsive cubosomes comprise bicontinuous cubic lipid membrane cores coated with pH-sensitive polyelectrolytes.^11–14^ They offer enhanced gastrointestinal (GI) delivery (Figure 1). The internal architecture of the cubosomes, constituted by curved lipid bilayers, provides a high interfacial area for drug encapsulation.^15–18^ The polyelectrolyte shell ensures stability in acidic stomach conditions. It triggers payload release at neutral intestinal pH.^12^ This design may overcome payload degradation and poor bioavailability.

**Figure 1:**
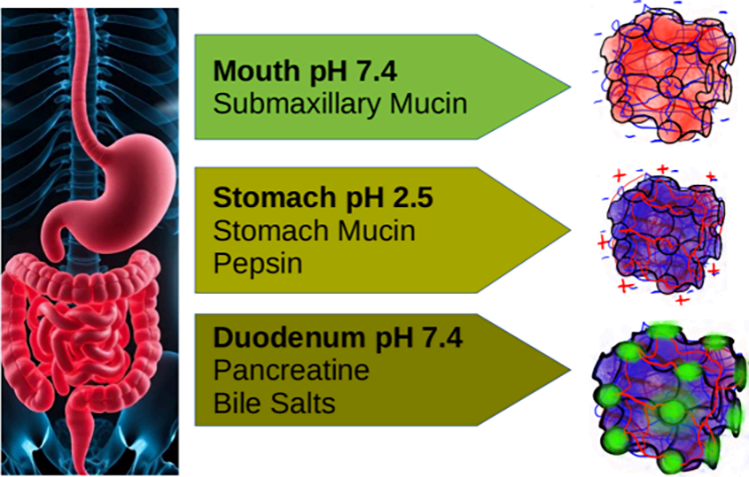
Interaction of intestinal enzymes and bile salts with polyelectrolyte shell (PS)-cubosomes in the gastrointestinal (GI) medium.

The polyelectrolyte shells may enhance the structure/performance features of stimuli-responsive materials.^19–21^ Remarkably, the charge density, ionization degree, molecular conformation, and hydrophilic/hydrophobic characteristics all contribute to polyelectrolytes to perform a myriad of biological interactions, even providing suitable mechanical properties for enhancing drug delivery.^22,23^ Indeed, stimuli yielded, for instance, by pH variation and specific ionic strength create feasibility for predictable structural changes in nanoparticles for controlled release of carried bioactives.

Despite this potential, the successful translation of such advanced materials requires a deep understanding of their dynamic behavior *in situ*. The GI tract presents a multifaceted barrier to drug delivery (Figure 1). It is characterized not only by drastic pH shifts (pH 1–3 in the stomach to pH 6–7.5 in the intestine) but also by a complex biomolecular milieu. This milieu comprises digestive enzymes (lipases, proteases), bile salts, phospholipids, and mucins.^24–26^ All these components interact dynamically with administered nanomaterials. Such interactions can alter surface properties via biomolecular corona formation. They can also induce structural rearrangements through enzymatic lipid digestion or surfactant effects.^27,28^ Ultimately, these interactions dictate payload release kinetics and absorption pathways. For digestible LNPs like monoolein-based cubosomes, the enzymatic generation of fatty acids and monoglycerides can drive complex self-assembly transitions (e.g., leading to induction of various micellar, vesicular, or other liquid crystalline phases).^29,30^ This process is critical to drug solubilization and transport but highly depends on the surrounding environment. Therefore, predicting and controlling the fate of functionalized LNPs necessitates moving beyond static characterizations. For this reason, we performed time-resolved structural measurements with designed advanced nanoarchitectures.

Acemannan, a bioactive polysaccharide from *Aloe vera*, was chosen as a challenging macromolecule for oral delivery. It possesses significant therapeutic potential, including immunomodulatory and wound-healing properties.^31–33^ However, it requires sophisticated delivery systems for oral efficacy due to stability concerns. pH-responsive polyelectrolyte-coated cubosomes were created here from monoolein, Pluronic F127, and combined chitosan-N-arginine/alginate shells that offer tailored nanostructured vehicles for encapsulation and controlled GI transit.^11,12^ However, the precise structural evolution of acemannan-loaded, functionalized cubosomes when exposed to specific GI components under relevant pH conditions remains largely unexplored. Understanding how these precisely engineered nanostructures respond to enzymatic activity (pepsin, pancreatin), surfactant action (bile salts), and bioadhesion (mucins) at both gastric and intestinal pH is critical for optimizing their performance.

The present study aims to address this knowledge gap. We employed *in situ* synchrotron small-angle X-ray scattering (SAXS) for high-resolution structural analysis of the fate and disassembly of advanced liquid crystalline materials in simulated biological environments. SAXS is a powerful technique capable of unambiguously detecting and identifying nanoscale structural transformations in real time.^27,34^ Here, it provides an in-depth spatiotemporal analysis of the topological phase behavior of acemannan-loaded, pH-responsive polyelectrolyte-shell cubosomes. We systematically investigated the structural integrity and phase transitions at pH 2.5 and pH 7.4. This was done upon cubosome exposure to key individual or mixed GI components. The influence of varying acemannan loading concentrations on these structural dynamics was also assessed. We hypothesized that the unique combination of environmental pH, specific biomolecular interactions, and payload concentration would induce distinct, quantifiable topological shifts in the cubosome nanostructure and its transformation or disassembly. The obtained fundamental insight into the dynamic structure/GI-medium interplay is essential for the rational materials engineering of next-generation LNPs. The goal is to achieve predictable and efficient performance of the self-assembled nanocarriers in the challenging gastrointestinal tract.

## 2 Results

We develop functionalized cubosomes using monoolein (1-oleoyl-rac-glycerol) as the primary lipid component, stabilized by Pluronic F127, and coated with pH-responsive polymers such as chitosan-N-arginine and alginate (Figure 2). These polysaccharides have been studied for their ability to modulate drug release in response to pH variations.^35,36^ Functionalized cubosomes may exhibit distinct mesophase topologies, including *Im3m* (primitive cubic), *Pn3m* (double diamond), and *Ia3d* (gyroid), depending on the physicochemical interactions between the bioactive compounds, the lipid bilayer, the polymer shells (chitosan-N-arginine/alginate complex), and the medium.^12^ These interactions might also induce a transformation into an inverted hexagonal (H_II_) phase under specific conditions. By monitoring these transformations with small-angle X-ray scattering (SAXS), complemented by Cryo-TEM imaging, we aim to elucidate the mechanisms governing the stability, potential disassembly pathways, and release characteristics of acemannan from these advanced delivery systems under physiologically relevant stresses.

**Figure 2:**
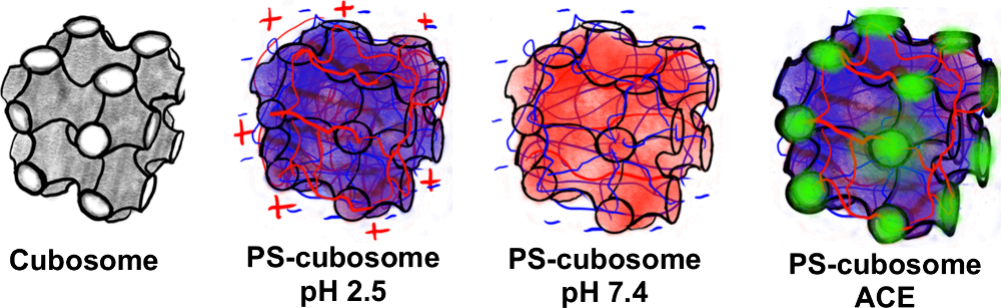
Different types of cubosomes with increasing complexity of core–shell nanoarchitecture, composed of cubosome lipid core and polyelectrolyte shell (PS) that is sensitive to pH of the GI environment and is of interest for acemannan (ACE) loading and transport.

### 2.1 Structures of Cubosomes and Polyelectrolyte-Shell Cubosomes as pH-Responsive Nanocarriers for Acemannan Encapsulation

We previously investigated hydrodynamic diameters of cubosomes, PS-cubosomes, and PS-cubosomes loaded with acemannan.^11,12^ As concluded from our studies, blank cubosomes present a colloidal size of around 247 ± 46 nm at pH 2.5 and 331 ± 63 nm at pH 7.4. PS-cubosomes had a slight increment to around 280 ± 65 nm at pH 2.5 and a significant size increase to 685 ± 107 nm at pH 7.4. This size increase was due to the pH-responsiveness of the polyelectrolyte shell, as discussed before.^11^ Then, the encapsulation of acemannan in PS-cubosome did not significantly change the colloidal features at pH 2.5. However, at pH 7.4, an increased surface-lamellarity on the cubosomes slightly increased the average size and caused some shape changes of the cubosomes, besides providing a partial phase transition.^12^ In the present study, the nanoorganization of cubosome formulations was investigated by SAXS for different configurations at pH 2.5 and pH 7.4. Figure 3 presents the SAXS profiles for blank monoolein/Pluronic F127 cubosomes (blue line), cubosomes coated with the chitosan-N-arginine/alginate polyelectrolyte shell (PS-cubosomes, light blue line), and PS-cubosomes loaded with 10% (red line), 20% (pink line), and 30% (brown line) acemannan relative to monoolein weight. In all patterns, distinct Bragg reflections were observed, indicated by arrows. The positions of these peaks mostly followed the ratio √2:√4:√6:√8:√10:√12. These are characteristic reflections corresponding to the (110), (200), (211), (220), (310), and (222) planes of a primitive cubic lattice of the *Im3m* space group. The *Im3m* cubic phase is typically formed upon dispersion of monoolein/Pluronic mixtures in excess water. The calculated lattice parameters (a_Q_) are presented in Table 1.

**Figure 3:**
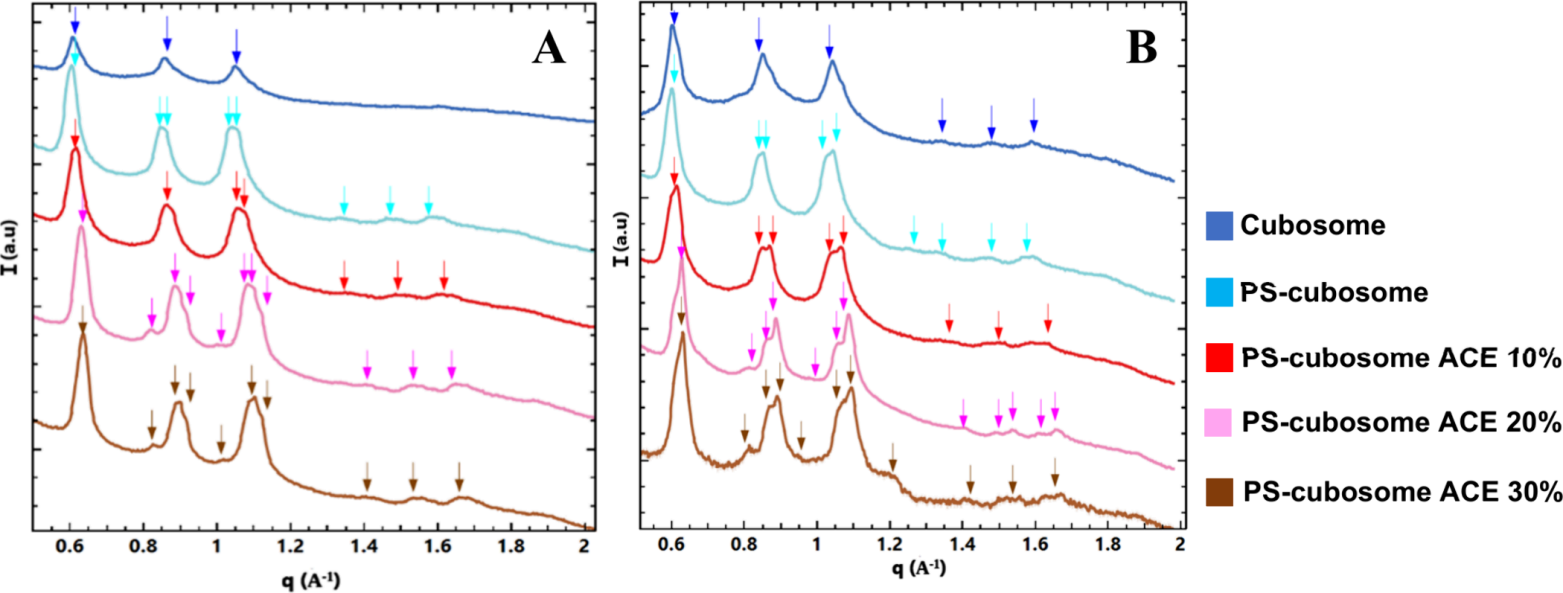
SAXS patterns, intensity (*I*) versus wavevector (*q*), at pH 2.5 (A), pH 7.4 (B), for blank monoolein/Pluronic F127 cubosomes (upper blue line), polyelectrolyte-shell cubosomes without acemannan (PS-cubosomes, light blue line), polyelectrolyte-shell cubosomes with 10% acemannan (red line), polyelectrolyte-shell cubosomes with 20% acemannan (pink line), and polyelectrolyte-shell cubosomes with 30% acemannan (brown line). Bragg peaks for identified cubic phases are indicated by arrows on top of the curves.

**Table 1:** Cubic Lattice Parameters, a_Q_, of Cubosomes Dispersed in Media with pH 2.5 and 7.4

sample	space group	lattice parameter (nm) pH 2.5	lattice parameter (nm) pH 7.4
cubosome	*Im3m*	14.70	14.92
PS-cubosome	*Im3m*	14.49–14.70	15.10–15.15
PS-cubosome 10% ACE	*Im3m*	14.49–14.70	14.66–14.90
PS-cubosome 20% ACE	*Im3m*	15.62–16.39–16.66	13.96–14.30–14.40
PS-cubosome 30% ACE	*Im3m*	13.69–14.28–14.43	13.73–14.12–14.22

The SAXS patterns of blank cubosomes (blue line, Figure 3) showed relatively sharp peaks, indicative of a well-ordered *Im3m* cubic structure. The lattice parameter a_Q_ was 14.70 nm at pH 2.5 and 14.92 nm at pH 7.4. Upon addition of the polyelectrolyte shell (PS-cubosomes, light blue line), particularly evident at pH 2.5 (Figure 3A), a broadening and splitting of some peaks occurred (e.g., around q corresponding to √4 and √6 positions, labeled q_2_ and q_3_). This suggested the induction of structural heterogeneity in the cubic lattice domains upon polymer shell (chitosan-N-arginine/alginate) coating. The lattice parameters, a_Q_, for PS-cubosomes ranged from 14.49 to 14.70 nm at pH 2.5, and 15.10 to 15.15 nm at pH 7.4, indicating slight variations (Table 1). Indeed, upon the addition of polyelectrolytes, the cubic size changes due to interactions between the different polymers in the medium.

The incorporation of acemannan into PS-cubosomes further modulated the structure, particularly at higher loading concentrations. At pH 2.5 (Figure 3A), as acemannan concentration increased, the heterogeneity or splitting of peaks became more pronounced. For PS-cubosomes with 10% acemannan, the lattice parameter, a_Q_, showed a slight increase compared to blank PS-cubosomes, ranging from 14.49 to 14.70 nm. With 20% acemannan, the lattice parameters increased further to 15.62 nm–16.39 nm–16.66 nm, suggesting swelling. However, with 30% acemannan, the lattice parameters decreased to 13.69, 14.28, and 14.43 nm, indicating compaction (Table 1). This behavior may be attributed to the nature of acemannan, a polymer primarily composed of repeating β-(1→4) acetylated mannose units, along with β-(1→4) glucose and α-(1→6) galactose. The number of these groups along the biopolymer chain determines its hydrophobic behavior, suggesting that acemannan may exhibit amphipathic properties.^37,38^ This allows it to interact with both the lipid chains of the cubosome membrane and the polyelectrolytes in its outer shell, leading to the formation of cubosomes of varying sizes. To better understand the mechanism by which acemannan interacts with the lipid and polymer components, we directly measured its surface activity using pendant drop tensiometry (see Supporting Information). The results demonstrated that acemannan is surface-active, causing a concentration-dependent decrease in the surface tension of the buffer solution. This confirms its amphiphilic character, which is crucial for its ability to partition at interfaces. The amphiphilic properties of acemannan provide a direct physical basis for its observed influence on the cubosome lattice structure, heterogeneity, and the swelling or compaction of the studied nanoparticles.

At pH 7.4 (Figure 3B), the peaks generally appeared sharper and better defined. For PS-cubosomes with 10% acemannan, lattice parameters (a_Q_) were 14.66 nm–14.90 nm. At 20% acemannan, they were 13.96 nm–14.30 nm–14.40 nm, and at 30% acemannan, they were 13.73 nm–14.12 nm–14.22 nm. Notably, at pH 7.4, peak splitting or heterogeneity extended to lower q values (around the √2 position, q_1_), which was less apparent at pH 2.5. Lipid cubosomes typically exhibited a smaller size compared to other formulations. However, upon the addition of polyelectrolytes and acemannan, their size increased due to interactions. The observed split peaks in q_3_ position for PS-cubosomes (Figure 3A), and in q_1_ at pH 7.4 (Figure 3B), particularly with acemannan, suggest distinct populations or internal domains within the cubosomes as related to pH sensitivity.

The high encapsulation percentage of acemannan, at an order of 90% as previously determined,^12^ certainly affects the lattice parameter splitting shown in Table 1, especially for 20 and 30% ACE.

### 2.2 Structural Analysis of SAXS Profiles Interacting with Submaxillary Mucin

The interaction between cubosome formulations and submaxillary mucin was investigated at pH 2.5 and 7.4. (Figure 4, Table 2). At pH 2.5, the SAXS profiles showed that the primary *Im3m* structure of the cubosomes was largely preserved (Figure 4A). No significant structural changes are detected compared to cubosomes without mucin. For blank cubosomes, the lattice parameter was 14.28 nm. For PS-cubosomes, a_Q_ was 14.92 nm. The samples still exhibit heterogeneity at different acemannan concentrations, but a slight shift in q values was noticeable as the acemannan concentration increased. With acemannan loading (10%, 20%, 30%), lattice parameters showed variations: 10% ACE (14.28 nm–14.43 nm), 20% ACE (16.36 nm–16.39 nm–16.66 nm), and 30% ACE (15.87 nm–16.33 nm–16.55 nm) (Table 2). A slight increase in scattering intensity at very low q values was observed, suggesting some mucin-induced network formation. The characteristic Bragg peaks of the *Im3m* phase remained, indicating that surface interactions did not cause structural transformation of the cubic phase core.

**Figure 4:**
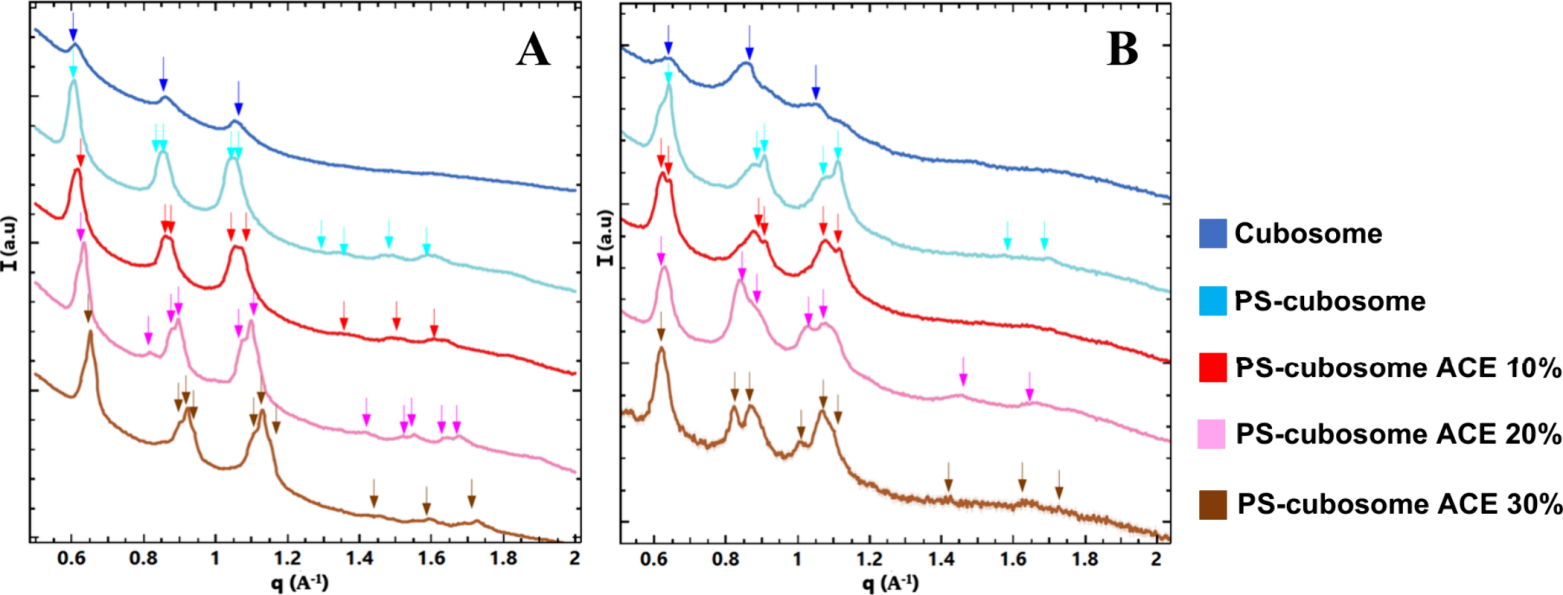
SAXS patterns of cubosomes after interaction with submaxillary mucin at (A) pH 2.5 and (B) pH 7.4. The color codes of the plots are the same as in Figure 3.

**Table 2:** Cubic Lattice Parameters, a_Q_, of Cubosomes Interacting with Submaxillary Mucin at pH 2.5 and 7.4

sample	space group	lattice parameter (nm) pH 2.5	lattice parameter (nm) pH 7.4
cubosome	*Im3m*	14.28	15.38
PS-cubosome	*Im3m*	14.92	14.34–14.43
PS-cubosome 10% ACE	*Im3m*	14.28–14.43	13.88–14.08–14.70
PS-cubosome 20% ACE	*Im3m*	16.36–16.39–16.66	12.19–14.28
PS-cubosome 30% ACE	*Im3m*	15.87–16.33–16.55	12.82–13.15

At pH 7.4 (Figure 4B), more significant changes in heterogeneity (split in the Braggs peaks) were observed, particularly for acemannan-loaded systems. The scattering intensities corresponding to acemannan-loaded nanostructures increased. Blank cubosomes had a lattice parameter of 15.38 nm. PS-cubosomes showed a_Q_ values of 14.34 nm–14.43 nm. For acemannan-loaded PS-cubosomes, the lattice parameters were: 10% ACE (13.88 nm–14.08 nm–14.70 nm), 20% ACE (12.19 nm–14.28 nm), and 30% ACE (12.82 nm–13.15 nm) (Table 2). The sample with 30% acemannan showed the most pronounced changes. A more evident peak splitting at each q vector was established, indicating a stronger differentiation between cubosome populations or the generation of internal domains. The overall *Im3m* phase was maintained, but its internal organization was substantially modulated by mucin interaction at this higher pH.

It should be noted that submaxillary mucin is fully protonated at pH 2.5, exhibiting greater hydrophobicity.^39^ As a result, its interaction with the nanoparticles seems uneven. The restricted binding to the outermost layer of the cubosomes affects the Bragg peak positions at this pH. In contrast, at pH 7.4, the mucin structure is in a state that allows stronger interaction with both the polyelectrolytes and the cubosome cores, leading to the structural changes observed at this pH.

### 2.3 SAXS Profiles of Cubosomes and Polyelectrolyte-Shell Cubosomes Interacting with Stomach Mucin

The SAXS patterns characterizing the cubosome-gastric mucin interaction (Figure 5, Table 3) show similarities to those of submaxillary mucin. Although both are mucin macromolecules, they differ in their interaction sites. For instance, submaxillary mucin contains a higher proportion of sialic acid compared to gastric mucin.^40^ This could significantly influence their mode of interaction with cubosomes. Previous studies have investigated the specific interaction of the two mucins with chitosan, a polymer known for its mucoadhesive properties.^40^ They have shown that submaxillary mucin has a more homogeneous interaction with chitosan. As a consequence, it may attain greater integration within cubosomes coated by a polyelectrolyte shell of this polysaccharide. Efficient integration may lead to increased structural heterogeneity of the cubosomes when interacting with submaxillary mucin.

**Figure 5:**
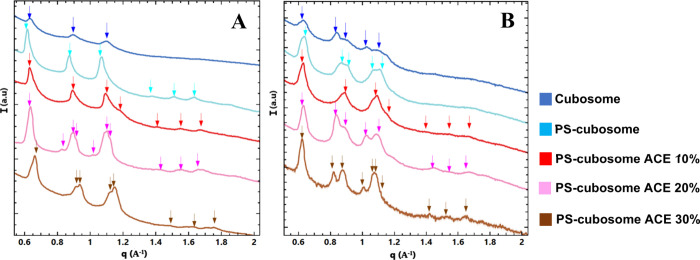
SAXS patterns characterizing the interactions of cubosomes with stomach mucin at (A) pH 2.5 and (B) pH 7.4. The color codes of the plots are the same as in Figure 3.

**Table 3:** Lattice Parameters, a_Q_, of Cubosomes Interacting With Stomach Mucin at pH 2.5 and pH 7.4

sample	space group	lattice parameter (nm) pH 2.5	lattice parameter (nm) pH 7.4
cubosome	*Im3m*	14.28	13.15–13.38
PS-cubosome	*Im3m*	14.49	13.88–15.62
PS-cubosome 10% ACE	*Im3m*	13.96	11.90
PS-cubosome 20% ACE	*Im3m*	15.62–16.39	13.51–13.81
PS-cubosome 30% ACE	*Im3m*	12.65–13.10–13.12	13.56–14.08

The results at pH 2.5 (Figure 5A, Table 3) indicated that gastric mucin did not cause major perturbation of the *Im3m* cubic phase lattice order, similar to submaxillary mucin. The lattice parameter for blank cubosomes was 14.28 nm. For PS-cubosomes, a_Q_ was 14.49 nm. Acemannan-loaded systems showed a_Q_ values of 10% ACE (13.96 nm), 20% ACE (15.62 nm–16.39 nm), and 30% ACE (12.65 nm–13.10 nm–13.12 nm) (Table 3). The Bragg peak intensities were generally lower compared to those for submaxillary mucin interactions, and the heterogeneity appeared less pronounced. This limited interaction in acidic media (pH 2.5) can be attributed to the structural reorganization of mucin in low pH environments, where it adopts a more compact and hydrophobic configuration, thereby reducing its affinity for mucoadhesive materials such as the polymers present in the polyelectrolyte shell.

Under alkaline conditions (pH 7.4), increased heterogeneity (coexistence of domains of different organization) was observed, similar to submaxillary mucin, especially for PS-cubosomes loaded with acemannan (Figure 5B). At pH 7.4, the gastric mucin structure becomes more extended and flexible, promoting interaction with accessible domains. This conformational change favored greater interaction with PS-cubosomes loaded with acemannan, increasing their structural heterogeneity, as observed in the SAXS profiles (Figure 5). Lattice parameters for blank cubosomes were 13.15 nm–13.38 nm. For PS-cubosomes, a_Q_ were 13.88 nm–15.62 nm. Acemannan-loaded systems showed values of 10% ACE (11.90 nm), 20% ACE (13.51 nm–13.81 nm), and 30% ACE (13.56 nm–14.08 nm) (Table 3). The differences determined from the Bragg peaks and their split are presented in Table 3. The *Im3m* cubic phase remained the dominant structure.

It can be suggested that the alkaline environment of the intestine will foster increased mucosal interaction, which correlates with the greater heterogeneity observed in PS-cubosomes loaded with acemannan under these conditions. Although gastric mucin exists in an acidic environment, in vivo studies have reported the presence of a pH gradient ranging from the acidic gastric lumen to more neutral areas near the apical surface of stomach epithelial cells.^41–43^ This may explain the reduced interaction observed in the more extreme acidic regions (Figure 5, Table 3). Given these circumstances, the interactions between the functionalized cubosomes and gastric mucin remained weak at pH 2.5.

### 2.4 SAXS Profiles of Cubosomes and Polyelectrolyte-Shell Cubosomes for Pepsin Interaction

The interaction of cubosomes with pepsin was investigated at both pH 2.5 and 7.4 (Figure 6, Table 4). At pH 2.5 (Figure 6A), where pepsin is enzymatically active,^44^ the SAXS profiles closely resemble those without pepsin. The characteristic cubic *Im3m* reflections were maintained across all formulations, and the peak heterogeneity associated with polyelectrolyte coating and acemannan loading persisted. Lattice parameters for blank cubosomes were 14.26 nm. For PS-cubosomes, a_Q_ = 15.10 nm. Acemannan-loaded systems showed a_Q_ values of 10% ACE (14.61 nm–14.79 nm), 20% ACE (14.20 nm–14.70 nm–14.79 nm), and 30% ACE (13.08 nm–13.51 nm), respectively (Table 4). This indicated that active pepsin did not significantly disrupt the core liquid crystalline structure.

**Figure 6:**
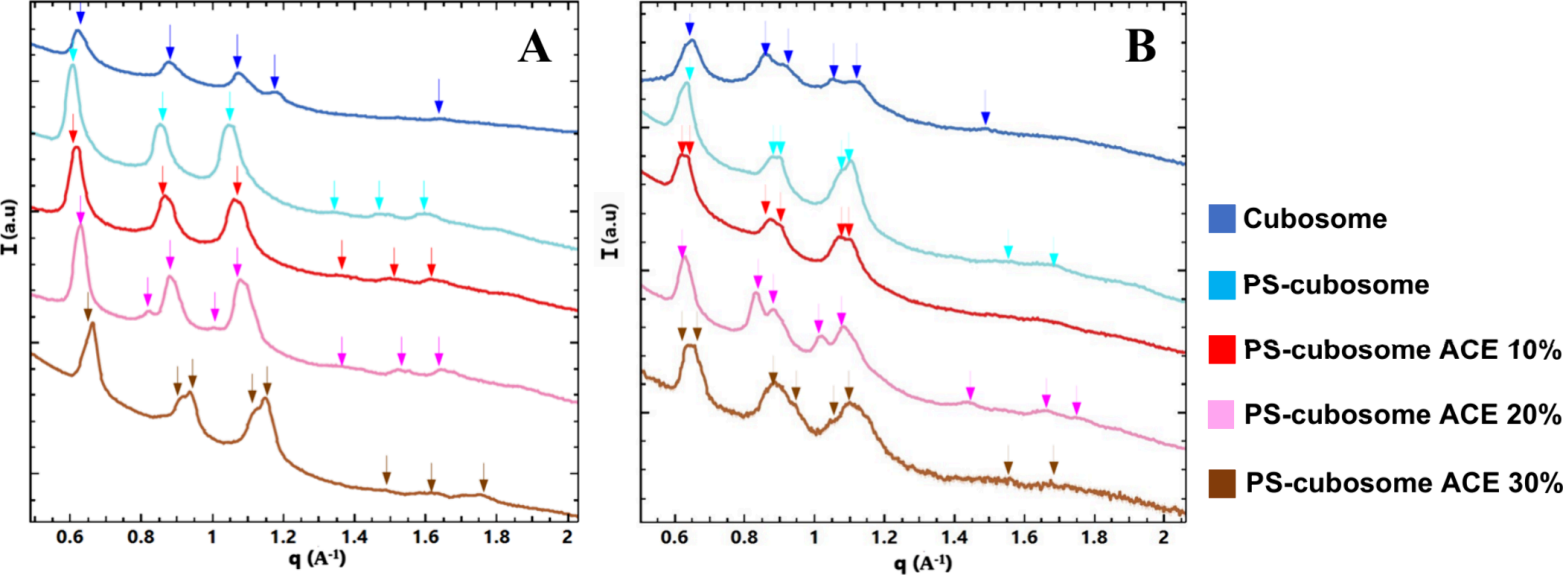
SAXS patterns after interaction of cubosomes with pepsin at (A) pH 2.5 and (B) pH 7.4. The color codes are the same as in Figure 3.

**Table 4:** Lattice Parameters, a_Q_, of Cubosomes Interacting with Pepsin at pH 2.5 and pH 7.4

sample	space group	lattice parameter (nm) pH 2.5	lattice parameter (nm) pH 7.4
cubosome	*Im3m*	14.26	13.00–13.17
PS-cubosome	*Im3m*	15.10	11.72–11.76
PS-cubosome 10% ACE	*Im3m*	14.61–14.79	13.53–14.12
PS-cubosome 20% ACE	*Im3m*	14.20–14.70–14.79	15.50–15.87
PS-cubosome 30% ACE	*Im3m*	13.08–13.51	16.94–17.85

In contrast, at pH 7.4 (Figure 6B), where pepsin is denatured,^45,46^ more noticeable changes occurred in the SAXS profiles compared to the initial structure in the absence of pepsin at pH 7.4 (Figure 3B). While the *Im3m* phase generally persisted, peak shapes and relative intensities were altered. The heterogeneity (peak splitting/broadening) appeared more expressed or pronounced. Lattice parameters for blank cubosomes were 13.00 nm–13.17 nm. For PS-cubosomes, a_Q_ were 11.72 nm–11.76 nm. Acemannan-loaded systems showed: 10% ACE (13.53 nm–14.12 nm), 20% ACE (15.50 nm–15.87 nm), and 30% ACE (16.94 nm–17.85 nm) (Table 4). The trend of lattice parameter variation with increasing acemannan concentration remained consistent at both pH values.

Pepsin is a protease active in the pH range of 1 to 5.5, maintaining its native structure at these pH values and preserving its proteolytic activity.^44^ However, when exposed to alkaline conditions, pepsin undergoes structural denaturation, leading to alterations in its biophysical properties.^45,46^ Our SAXS analyses after interaction with pepsin revealed notable changes in the PS-cubosomes at pH 7.4, with more pronounced heterogeneity compared to systems exposed to acidic media (Figure 6, Table 4). This implies that pH variations induce the exposure of hydrophobic regions in the structure of pepsin, thus enhancing its overall hydrophobic character. It is suggested that the increase in hydrophobicity favors the enzyme’s affinity for the cubosome membranes, facilitating interactions that affect the lattice size distribution. At acidic pH (2.5), pepsin presents a conformation where polar groups dominate its surface, giving it a positive charge and reducing its ability to disrupt the lipid membrane of the cubosome. Under this condition, it can interact superficially with the polymeric coating without inducing significant structural changes. At higher pH conditions, specific residues such as Glu4, Glu13, and Asp159 undergo deprotonation, leading to an increase in repulsive electrostatic interactions and resulting in a structural reorganization that ends in the denaturation of pepsin.^45,46^ This reorganization exposes more apolar regions, enhancing its ability to insert into the cubosome membrane or directly interact with acemannan. Such a phenomenon would explain the greater structural heterogeneity observed in PS-cubosomes at pH 7.4 (Figure 6), further supporting the idea that pH-induced conformational changes in pepsin have a direct impact on interactions with cubosome systems. These findings emphasize the importance of considering pH-dependent structural transformations of digestive enzymes and system components in the design of environmentally responsive drug delivery systems.

### 2.5 Structural Changes of Cubosomes and Polyelectrolyte-Shell Cubosomes Interacting with Bile Salts

The interaction with bile salts showed dramatic pH-dependent effects (Figure 7). At pH 2.5, bile salts induced significant structural changes (Figure 7A). The Bragg peaks of the initial *Im3m* cubic structure disappeared in most cases. For PS-cubosomes (light blue line), distinct scattering peaks were observed at q_1_ = 1.22 Å^–1^ and q_2_ = 2.44 Å^–1^. Similar peaks, but slightly shifted, were seen for PS-cubosomes with 10% acemannan (red line: q_1_ = 1.22 Å^–1^, q_2_ = 2.44 Å^–1^) and 20% acemannan (pink line: q_1_ = 1.16 Å^–1^, q_2_ = 2.34 Å^–1^). These peak positions, spaced in a 1:2 ratio, are characteristic of a lamellar (Lα) phase. However, for nonfunctionalized (blank) cubosomes and PS-cubosomes with 30% acemannan (brown line), no clear lamellar phase formation was found. Instead, broad scattering features suggested intermediate structures or vesicle formation upon cubosome disassembly.

**Figure 7:**
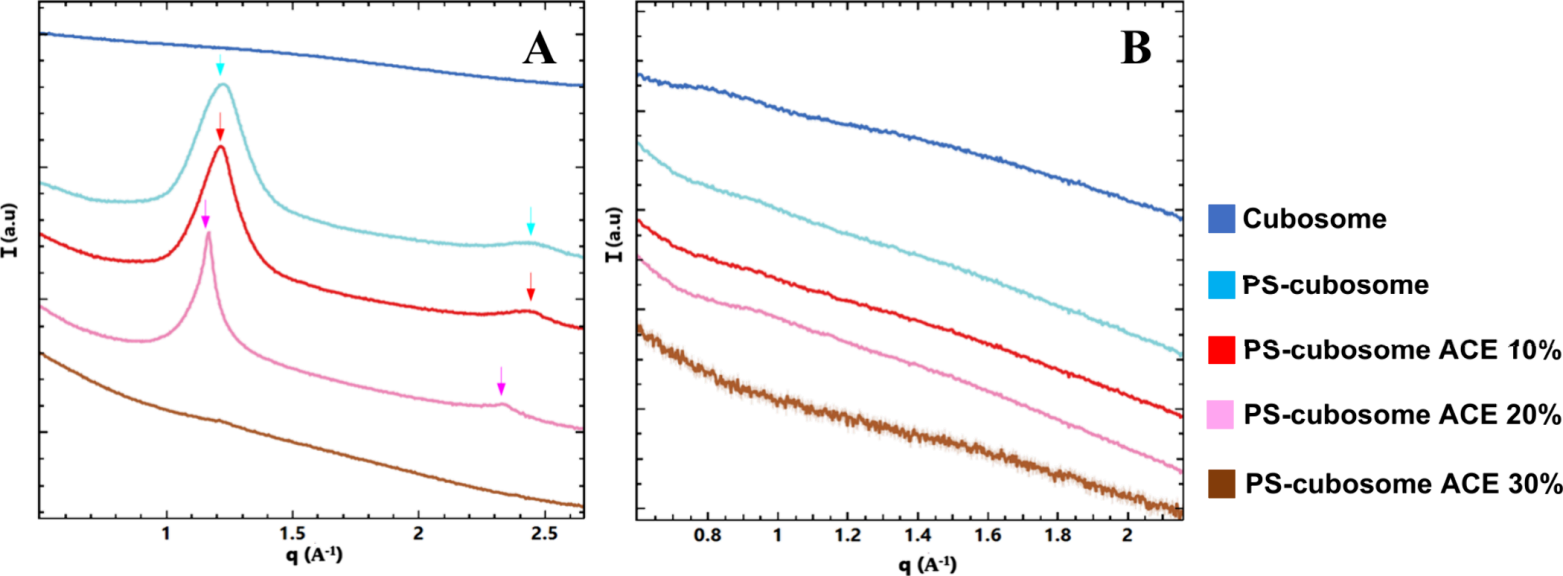
SAXS patterns revealing dramatic structural changes upon the interaction of cubosomes with bile salts at (A) pH 2.5 and (B) pH 7.4. The color codes are the same as in Figure 3.

Conversely, at pH 7.4 (Figure 7B), adding bile salts resulted in the complete disappearance of Bragg peaks associated with the *Im3m* cubic phase across all formulations. The resulting SAXS profiles were dominated by broad scattering features characteristic of bile salt aggregates, indicating complete solubilization of the cubosome structure. No detectable lamellar or other ordered phases were observed in any sample at this pH.

Bile salts play a crucial role in the solubilization and absorption of lipids in the intestinal tract.^47–49^ Acting as natural surfactants, they facilitate lipid emulsification.^50^ This effect promotes efficient absorption during digestion. At low pH, their interaction with chitosan can lead to the formation of micelle-like clusters that influence the structural transitions of the cubosomes.

### 2.6 SAXS Profiles of Cubosomes and Polyelectrolyte-Shell Cubosomes Interacting with Pancreatin

Pancreatin represents a more complex enzymatic system, composed of amylase, lipase, and protease, each with specific functions. Amylase catalyzes the degradation of starches, lipase acts on triglycerides, and protease cleaves peptide bonds.^51–53^ These enzymes work in coordination in the duodenum, in a typically alkaline environment, which is relevant for stability and functionality studies of cubosomes under simulated gastrointestinal conditions. The performed SAXS study demonstrated that interactions with pancreatin also display a strong pH dependence (Figure 8, Table 5). At pH 2.5 (Figure 8A), where pancreatic lipase has negligibly low activity, the SAXS profiles remained largely unchanged compared to the initial cubosomes (see cubosome structures without pancreatin interaction, Figure 3A). The characteristic *Im3m* cubic phase reflections were retained. Lattice parameters for blank cubosomes were a_Q_ = 14.49 nm and for PS-cubosomes, a_Q_ = 14.92 nm. Acemannan-loaded systems showed a_Q_ values of 10% ACE (14.28 nm), 20% ACE (15.62 nm–16.15 nm–16.39 nm), and 30% ACE (16.12 nm–16.66 nm) (Table 5). The repeat spacing increased as the acemannan concentration increased, but the cubic phase symmetry persisted.

**Figure 8:**
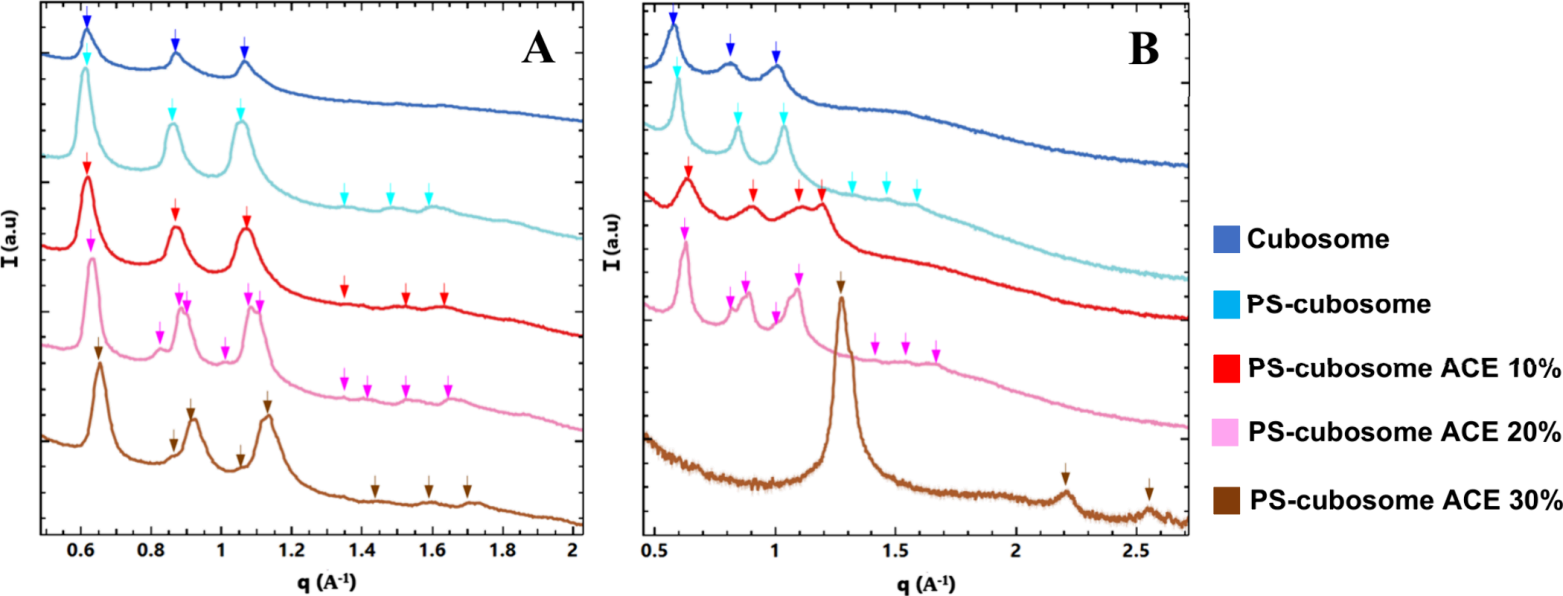
SAXS patterns characterizing the interaction of cubosomes with pancreatin at (A) pH 2.5 and (B) pH 7.4. The color codes are the same as in Figure 3.

**Table 5:** Lattice Parameters of Cubosomes, a_Q_, or Hexosomes, a_HII_, after Interaction with Pancreatin at pH 2.5 and pH 7.4

sample	space group	lattice parameter (nm) pH 2.5	lattice parameter (nm) pH 7.4
cubosome	*Im3m*	14.49	15.38
PS-cubosome	*Im3m*	14.92	14.49
PS-cubosome 10% ACE	*Im3m*	14.28	15.62–19.23
PS-cubosome 20% ACE	*Im3m*	15.62–16.15–16.39	15.87–16.66–16.94
PS-cubosome 30% ACE	*Im3m*/H_II_	16.12–16.66	5.00

Profound structural transformations occur at pH 7.4 (Figure 8B), where pancreatic lipase is highly active. The initial *Im3m* Bragg peaks rapidly diminished or disappeared. For blank cubosomes (lattice parameter a_Q_= 15.38 nm) and PS-cubosomes (a_Q_ = 14.49 nm), as well as PS-cubosomes with 10% ACE (15.62 nm–19.23 nm) and 20% ACE (15.87 nm–16.66 nm–16.94 nm), the SAXS profiles showed appearance of peaks consistent with a lamellar (Lα) phase (peaks spaced in the ratio 1:2:3···). They sometimes coexisted with broader features (Figure 8B).

Most strikingly, for PS-cubosomes containing 30% acemannan, a phase transition from cubic to an inverted hexagonal (H_II_) phase occurred. This was evidenced by Bragg reflections with ratios 1:√3:√4. The calculated lattice parameter for this H_II_ phase was approximately 5 nm (Table 5, bottom right). The H_II_ phase transition was unique to the 30% acemannan formulation at pH 7.4.

To further investigate the pancreatin-induced H_II_ phase transition occurring at pH 7.4 with PS-cubosomes with 30% acemannan, real-time (RT-SAXS) measurements were performed (see the results in Section 2.8).

### 2.7 Structural Fate of Cubosomes and Polyelectrolyte-Shell Cubosomes Interacting with Duodenum Mixture

Figure 9 shows the SAXS results characterizing the interaction of cubosomes with a simulated duodenum mixture (containing pepsin, pancreatin, and bile salts).^54^ The data suggest that bile salts are the primary contributors to cubosome disassembly at pH 7.4. The interaction between the proteins and the cubosome membrane may facilitate disassembly while stabilizing the lamellar structure. At pH 2.5 (Figure 9A), the SAXS profiles for acemannan-loaded PS-cubosomes (10%, 20%, 30%) showed the induction of lamellar phases. For 10% acemannan, two lamellar phases appeared to coexist. With 20% acemannan, multiple lamellar phases were evident. At 30% acemannan, the formation of lamellar or H_II_ phases is suggested but not well-resolved by SAXS alone. In contrast, PS-cubosomes without acemannan exposed to this mixture retained their cubic phase organization. This indicated that acemannan, under the acidic conditions of the mixture, promotes a transition to lamellar structures.

**Figure 9:**
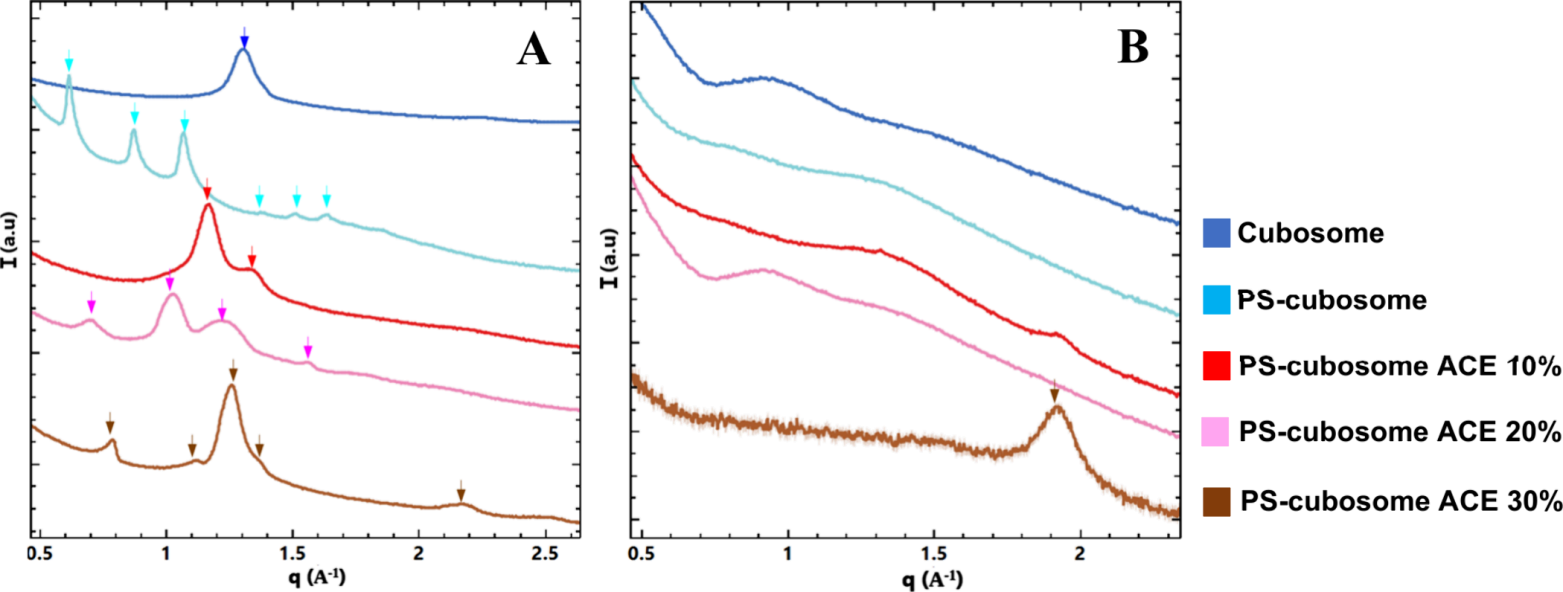
SAXS patterns for cubosome interaction with a duodenum mixture (containing pepsin, pancreatin, and bile salts) at (A) pH 2.5 and (B) pH 7.4. The color codes of the plots are the same as in Figure 3.

At pH 7.4, the SAXS patterns showed a complete disappearance of the *Im3m* Bragg peaks for all formulations (Figure 9B). The profiles were dominated by broad scattering, similar to the effect of bile salts alone at pH 7.4 (Figure 7B) and pancreatin alone (Figure 8B, for lower acemannan concentrations). This indicates rapid and complete disassembly of the original cubosome nanostructure into mixed micelles or digestion-product phases.

### 2.8 Real-Time Small-Angle X-ray Scattering (RT-SAXS) Investigation

The structural evolution of PS-cubosomes functionalized with 30% acemannan in the presence of the enzyme pancreatin at pH 7.4 was monitored through time-resolved SAXS pattern acquisition, as shown in Figure 10. The interaction between the components led to notable topological changes, indicating progressive structural transitions over time.

**Figure 10:**
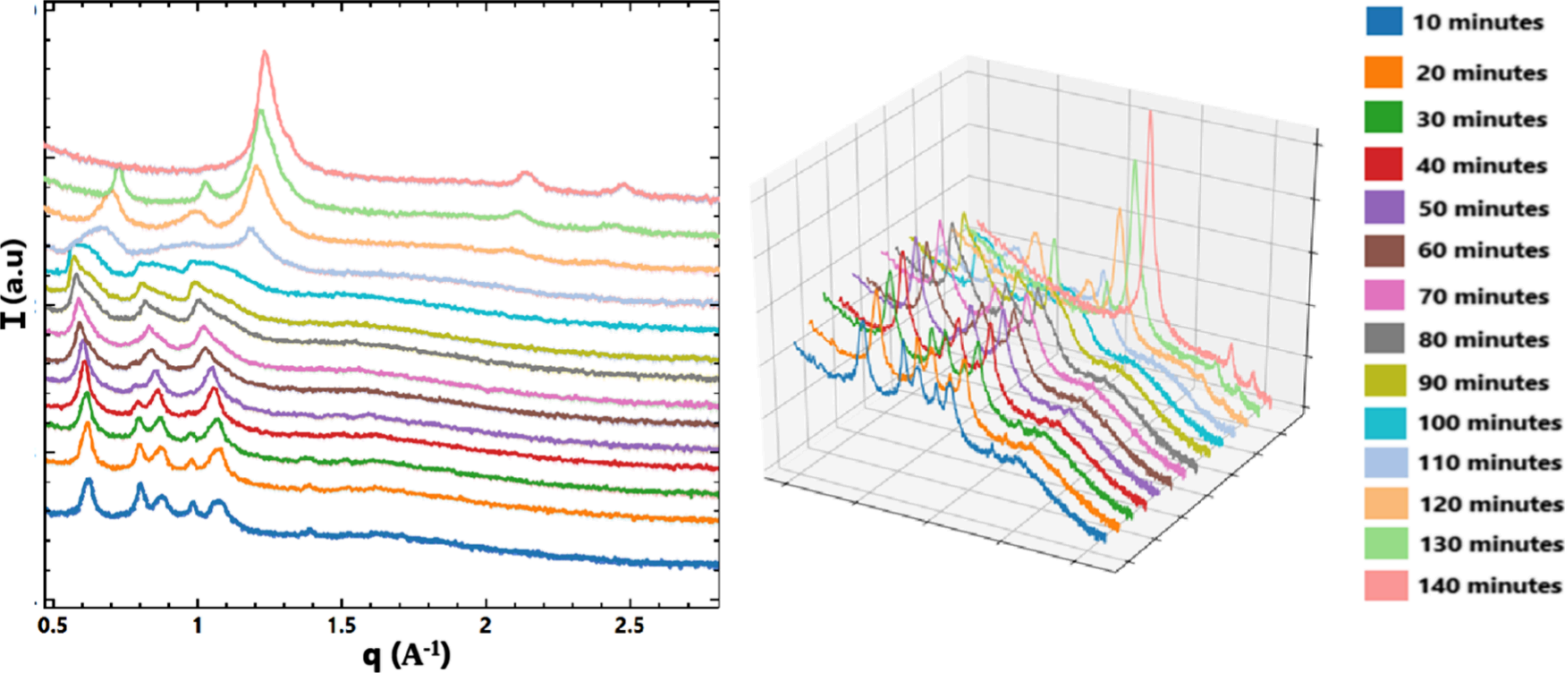
Real-time SAXS patterns recorded upon the interaction of PS-cubosomes (with 30% acemannan) and pancreatin at pH 7.4. The time points (minutes) are indicated for every curve. A 3D plot of the acquired sequence is presented with the same color codes.

In the early stages of the interaction (within the first 40 min), the scattering profiles revealed well-defined split peaks at the q_2_ and q_3_ positions of the *Im3m* phase. This indicated the coexistence of two distinct cubosome populations with slightly different internal lattice parameters (a_Q_). As the interaction progressed, particularly after the first 40 min, these distinct populations began to merge. This reflected the gradual attenuation and eventual merging of the split peaks. The transformation process was initiated by the disappearance of the double peak at q_3_, followed by the merging of the q_2_ peak at approximately the 60 min time point. The transition culminated in the formation of a uniform liquid crystalline phase at around 60–90 min (Figure 10).

Following the peak recovery of the nonlamellar phase, at 90–110 min elapsed time, an increase in the Bragg peak intensities was established. It indicated greater structural heterogeneity, which was not associated with a single dominant population. Finally, starting at approximately t = 120 min, an increase in the intensity of the peaks corresponding to an inverted hexagonal H_II_ phase was observed. This transformation was marked by an intensive q_1_ peak (the primary (10) reflection of the H_II_ phase), associated with a complete formation of the H_II_ phase after 120 min (Figure 10). Thus, the acquired RT-SAXS data provided a detailed kinetic pathway of the *Im3m* → H_II_ transition provoked by the cubosome-pancreatin interaction.

### 2.9 Cryo-TEM Imaging after Interaction between PS-Cubosomes (30% Acemannan) with a Duodenum Mixture or Pancreatin

Cryo-TEM was used to investigate the nanostructures resulting from specific interactions that yielded significant structural features in the SAXS patterns.

#### PS-Cubosomes (30% Acemannan) and a Duodenum Mixture

Control PS-cubosomes with 30% acemannan (no duodenum mixture) showed a well-defined cubic morphology (Figure 11A). The cubic *Im3m* inner structure was confirmed by Fourier transform analysis (inset in Figure 11A). Formation of irregular vesicular shapes or faceted lamellar-phase structures was observed after interaction with the duodenum mixture at pH 2.5 (Figure 11B). This result corroborated with the SAXS data (Figure 9A) and confirmed that the duodenum mixture disassembles the cubosomes into lamellar bilayer membranes at acidic pH. At pH 7.4 (Figure 11C), the interaction with the duodenum mixture resulted in the formation of micelle-like aggregates throughout the sample. This aligns with the SAXS data (Figure 9B), which showed complete disassembly of the lipid membrane architectures into micellar structures.

**Figure 11:**
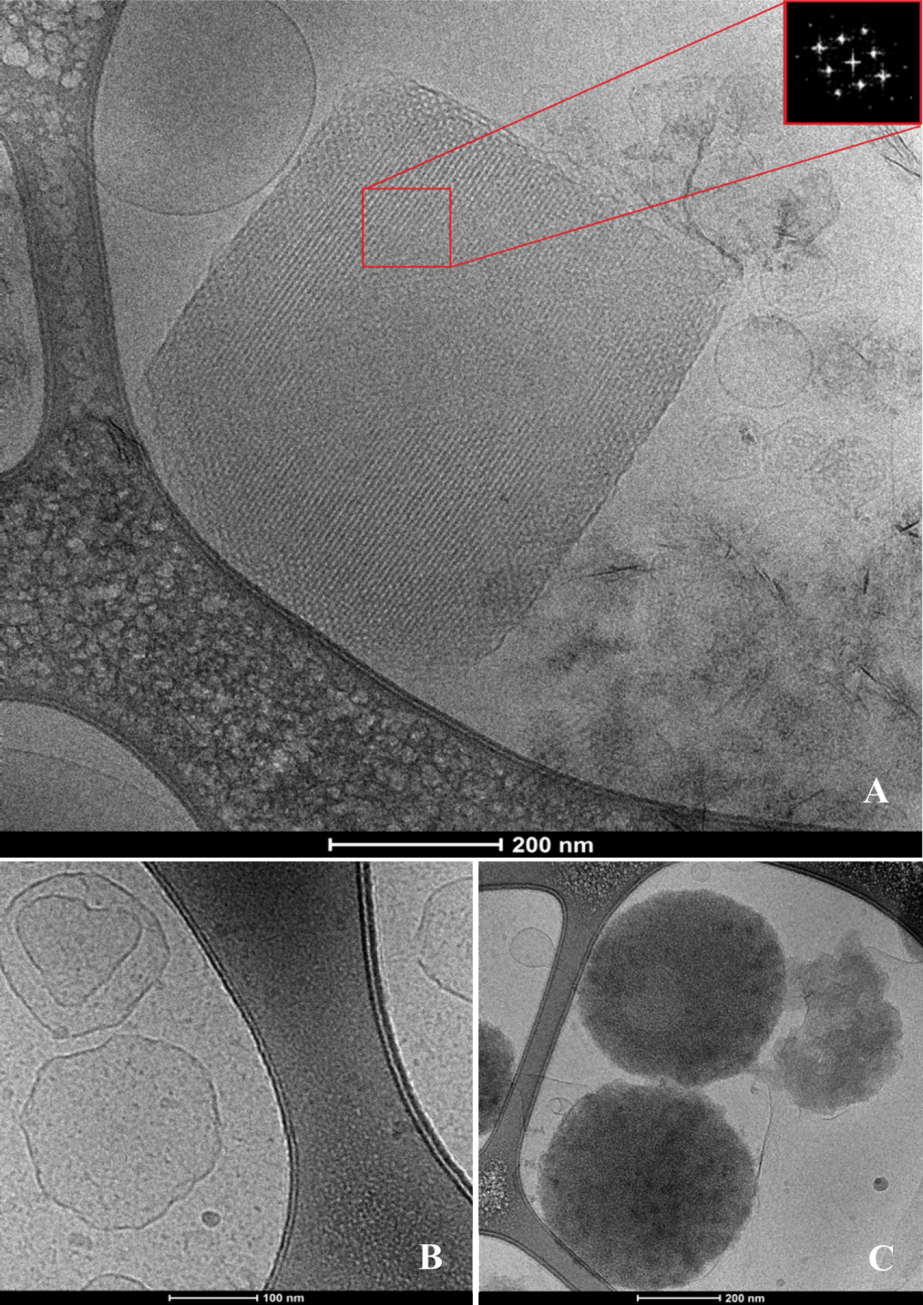
Cryogenic electron microscopy to PS-cubosome upon interaction with a duodenum mixture (containing pepsin, pancreatin, and bile salts) at pH 2.5 and 7.4. (A) Control PS-cubosomes with 30% acemannan (no interaction with the duodenum mixture). (B) PS-cubosomes with 30% acemannan after interaction with the duodenum mixture at pH 2.5 and (C) at pH 7.4.

The generated micelles may interact with acemannan and with the polymers coating of the cubosomes, acting as emulsifying agents. In doing so, they can stabilize the surface of the newly formed micelles. The latter may further influence the bioavailability and transport of the acemannan formulations in physiological environments.

#### PS-Cubosomes (30% Acemannan) and Pancreatin (pH 7.4)

Control samples of PS-cubosomes with 30% acemannan (Figures 12A) displayed a cubic liquid crystalline structure of the characteristic *Im3m* cubic space group symmetry. An onset of a phase transition was detected immediately after initiating the contact with pancreatin at pH 7.4 (Figures 12B, time 0.5 min). The Cryo-TEM images showed regions of disrupted cubic structure and emerging new phases. A Fourier transform analysis for symmetry identification was difficult due to multiple phase orientations. After 150 min of interaction (Figures 12C), the Cryo-TEM images revealed the complete formation of an inverted hexagonal phase (H_II_), characterized by arrays of aqueous cylinders packed in a lipid matrix. These morphological results strongly corroborate the RT-SAXS measurements (Figure 10). They fully confirmed the *Im3m* to H_II_ structural phase transition induced by pancreatin in the presence of 30% acemannan at pH 7.4.

**Figure 12:**
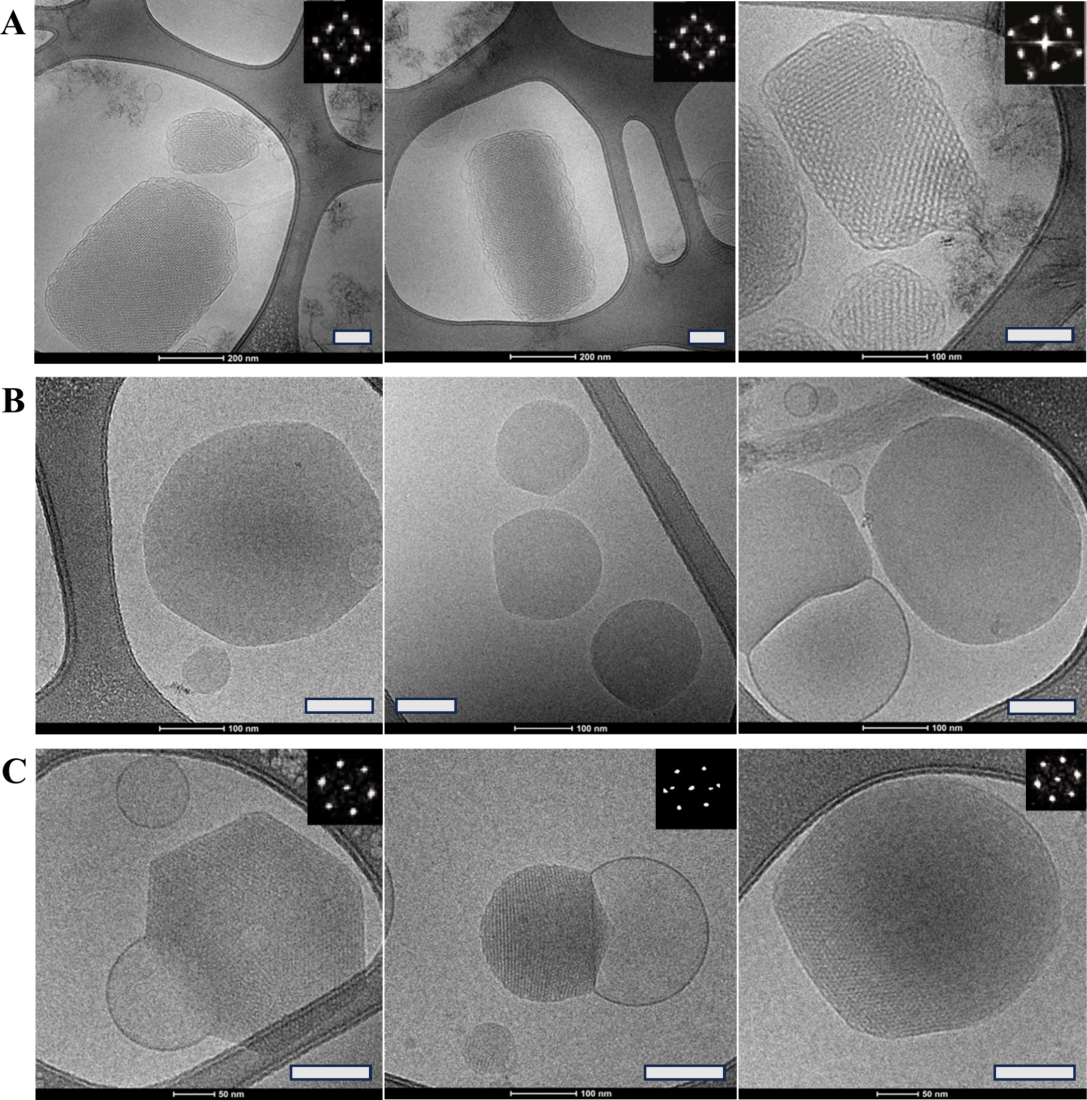
Cryo-TEM images of PS-cubosomes (containing 30% acemannan) interacting with pancreatin. (A) Control PS-cubosomes with 30% acemannan (no interaction with pancreatin). (B) PS-cubosomes (30% acemannan) at time 0.5 min interaction with pancreatin, and (C) topologies observed after 150 min interaction with pancreatin. For reference, all inserted white bars represent a span of 100 nm.

## 3 Discussion

The performed structural study advances beyond previous work, which primarily examined simpler lipid systems, bulk digestion, or equilibrium states.^8,27,28^ By attributing specific structural changes to individual molecular interactions under controlled conditions, our approach uncovers the intricate interplay between the nanocarrier’s hierarchical design (organized lipid core, pH-sensitive shell, bioactive payload, and its concentration), and the dynamic gastrointestinal environment. We performed a comprehensive analysis of the structural evolution of pH-responsive, acemannan-loaded polyelectrolyte-shell cubosomes under simulated GI conditions. The combination of SAXS and Cryo-TEM, including time-resolved studies, provided unprecedented insights into how the nanocarrier’s architecture and payload respond to the challenges posed by the simulated GI environment.

### 3.1 Impact of Functionalization and Polysaccharide Loading on Lipid Cubosome Structure

The structural characterization in Figure 3 revealed that both the polyelectrolyte shell (chitosan-N-arginine/alginate) and the encapsulated bioactive acemannan significantly modulate the *Im3m* cubic structure of monoolein cubosomes (Table 1). The shell-induced structural heterogeneity, evidenced by SAXS peak broadening and splitting, is likely due to interfacial interactions between the polymers and the lipid bilayer, altering local membrane curvature or hydration. This effect was more pronounced at pH 2.5, where chitosan is highly protonated. Acemannan, an amphiphilic polysaccharide,^37^ further complicated the internal structure in a concentration- and pH-dependent manner. Acemannan from *Aloe vera* is a pharmacologically active compound that possesses antioxidant, immunomodulatory, antibacterial, and regenerative properties.^32,33^ The observed swelling at 10% ACE loading and compaction at higher concentrations (20–30% ACE) suggest intricate partitioning behavior. Acemannan could reside in the aqueous channels, intercalate into the lipid bilayer, or interact with the polyelectrolyte shell.

The pH-dependent protonation of chitosan-N-arginine and alginate, alongside potential pH effects on acemannan itself, governs these interactions, leading to more pronounced heterogeneity at pH 7.4 compared to pH 2.5. Therefore, the structural heterogeneity can be attributed to a direct interaction between acemannan and the polyelectrolyte coating. Acemannan exhibits a variable degree of acetylation, which confers both hydrophobic and hydrophilic properties, giving it an amphiphilic character.^37^ (see the results in the Supporting Information). This feature may enhance its affinity for the cubosome’s lipid membrane and facilitate the formation of emulsifying microenvironments. Combined with the polyelectrolyte coating, the overall system demonstrates adaptive behavior in response to intestinal pH variations, contributing to the development of a controlled-release delivery platform. These detailed insights into payload-matrix-shell interactions within functionalized cubosomes advance our understanding beyond simpler lipid systems.

### 3.2 Structural Response and Stability in Simulated Gastric Conditions (pH 2.5)

Under acidic gastric conditions (pH 2.5), the coated cubosomes demonstrated remarkable stability against pepsin (Figure 6A) and mucins (Figures 4A, A). Active pepsin did not disrupt the core cubic *Im3m* structure, indicating the polyelectrolyte shell and lipid matrix are resistant to its proteolytic activity. Mucin interactions, mediated by the mucoadhesive chitosan shell,^55^ led to some surface aggregation but preserved the core cubic architecture. Mucin is a glycoprotein that facilitates the transport of the bolus to the stomach and is highly resistant to a wide range of pH conditions. Mucin’s compact structure at low pH likely limits deeper penetration.^42^ This resilience is crucial for protecting encapsulated cargo during gastric transit.

However, bile salts at pH 2.5, even as weakly surface-active protonated species,^24^ induced partial disassembly to lamellar (Lα) phases in formulations with 10–20% acemannan (Figure 7A). The absence of Lα phase in blank or highly loaded (30%) systems suggests a complex interplay where acemannan concentration modulates susceptibility to bile salt penetration. The simulated duodenum mixture at pH 2.5 also led to lamellar phase formation for acemannan-loaded systems (Figure 9A, Figure 12B), primarily driven by bile salts. These findings highlight that while the shell offers protection, payload characteristics can influence stability against specific gastric components.

### 3.3 Structural Transformations and Disassembly in Simulated Intestinal Conditions (pH 7.4)

The transition to neutral intestinal pH (pH 7.4) triggered significant structural changes, as intended by the pH-responsive design, and revealed the dominance of intestinal digestive factors. Mucin interactions at pH 7.4 (Figures 4B, B) were more pronounced, leading to greater heterogeneity. This is likely due to mucin’s extended conformation^42^ and altered shell properties (less charged chitosan, and swollen alginate),^56^ facilitating in-depth interactions. Denatured pepsin at pH 7.4 (Figure 6B) also induced minor structural perturbations, probably via hydrophobic adsorption, but did not cause disassembly.

The most dramatic effects were seen with bile salts and pancreatin. Bile salts at pH 7.4, acting as potent anionic surfactants,^24,47^ caused complete and rapid disassembly of all cubosome formulations into mixed micellar solutions (Figure 7B). This confirmed that the shell and payload cannot prevent solubilization by physiological concentrations of intestinal surfactants.

Pancreatin, containing active lipase,^53^ also led to the disappearance of the initial cubic phase (Figure 8B). For most formulations, intermediate lamellar phases formed, consistent with the generation of digestion products like oleic acid and residual monoolein.^25,30,48^ Remarkably, for PS-cubosomes with 30% acemannan, pancreatin induced a time-dependent *Im3m* to inverted hexagonal (H_II_) phase transition (Figures 8B,10

The detailed elucidation of the acemannan-concentration-dependent, pancreatin-induced cubic *Im3m*-to-H_II_ transition, supported by RT-SAXS and Cryo-TEM, is a key novel contribution, highlighting active payload participation in modulating enzymatic processes and nanostructural fate. The performed systematic, condition-dependent structural analysis bridges materials science and pharmaceutical nanotechnology, offering crucial insights for rational material design.

The simulated intestinal mixture (pH 7.4) confirmed the dominance of these digestive processes, leading to rapid and complete structural breakdown into micellar/digestion product phases (Figure 9B, Figure 11C).

### 3.4 Implications for Oral Drug Delivery

The reported structural insights have significant implications for designing pH-responsive cubosomes. Our previous studies have shown that in vitro payload release is pH-dependent, where in acidic solution, acemannan reached an average of 50% release after 6 h of dialysis. Instead, for the slightly alkaline solution, 68% release was attained after 28 h. Although the release profile was more attenuated in pH 7.4, a primarily Fickian diffusion was characterized for both in vitro conditions.^12^ Herein, including gastrointestinal components, the PS-cubosome demonstrated gastric stability (pH 2.5) against pepsin and mucin, supporting its potential for payload protection. However, susceptibility to bile salts and pancreatic lipase at intestinal pH (pH 7.4) indicates that payload release in the intestine will likely be governed by rapid lipid matrix breakdown, rather than solely by shell dissolution. The polyelectrolyte shell’s primary role appears to be gastric protection and possibly modulating mucoadhesion,^56^ while the intestinal environment dictates rapid transformation once the pH trigger is activated. The influence of acemannan concentration on the nanocarrier structure, stability against bile salts at low pH, and the unique H_II_ transition with pancreatin emphasizes that payload interactions must be carefully considered during formulation development. These findings provide a valuable mechanistic foundation for engineering advanced nanomaterials for effective oral drug delivery, considering the dynamic GI environment in its full complexity.

Overall, our study has made significant progress in the application of high-resolution structural analysis in understanding self-assembled delivery systems for oral delivery. While static SAXS has defined equilibrium phase diagrams^57,58^ and time-resolved studies have tracked bulk digestion kinetics,^27,48,49^ the present work dissects the effects of *individual* GI components under controlled pH conditions. This allowed the attribution of specific structural changes (e.g., disassembly by bile salts vs lipase or mucoadhesion by mucins) to distinct molecular interactions, providing a more mechanistic understanding than previously available. Furthermore, most prior *in situ* SAXS studies involved simpler lipid systems or conventional nanoparticles.^2,5,7,34^ The present investigation assessed a more advanced, hierarchical material comprised of a pH-responsive, polyelectrolyte-coated cubosome carrying a large polysaccharide payload. Characterizing how this complex architecture, and importantly, how payload concentration, influences the response to GI challenges, represents a novel application of SAXS in the development of advanced materials for oral delivery.

Finally, it is important to acknowledge the limitations of the current in vitro model. Our study provides a detailed molecular and nanoscale picture of component-specific interactions, but does not account for the full physiological complexity of the GI tract, which includes dynamic factors such as peristaltic motion, continuous mucus secretion and turnover, the presence of a cellular absorption barrier, and the gut microbiome. These factors could further influence the residence time, biomolecular corona formation, and ultimate fate of the nanocarriers. Therefore, while our findings provide a crucial mechanistic foundation, future in vivo studies will be essential to validate these behaviors and fully assess the therapeutic efficacy of these advanced delivery systems.

## 4 Conclusions

One key finding from the performed structural investigation of pH-responsive polyelectrolyte-shell cubosomes loaded with acemannan at varying concentrations is that the chitosan-N-arginine/alginate shell provides a protective function under gastric conditions (pH 2.5). In this environment, the cubosomes successfully maintained their structural integrity against the digestive enzymes pepsin and mucins. However, even at this acidic pH, bile salts could induce partial transitions to lamellar phases, a process influenced by acemannan concentration. Conversely, under simulated intestinal conditions (pH 7.4), the investigated system responded dynamically. While interactions with mucins and denatured pepsin caused only minor perturbations, bile salts led to the complete and rapid disassembly of all cubosome types into micellar structures. Pancreatin-induced enzymatic digestion also resulted in disassembly, notably triggering a time-dependent transition from the initial *Im3m* cubic phase to an inverted hexagonal (H_II_) phase for cubosomes loaded with 30% acemannan. This unique payload-concentration-dependent effect, corroborated with RT-SAXS and Cryo-TEM results, highlighted a synergistic role of acemannan in modulating enzyme access or activity. The simulated duodenum mixture studies confirmed these trends, leading to lamellar phases at pH 2.5 for acemannan-loaded systems and complete disassembly at pH 7.4, predominantly driven by bile salts. These fine structural responses and component-specific disassembly pathways provide crucial mechanistic insights. The achieved understanding is pivotal for the rational design of advanced nanocarriers, enabling controlled release and enhanced protection of sensitive bioactives for optimized oral drug delivery within the complex and dynamic environment of the GI tract.

Environmental pH plays a crucial modulatory role in the investigated interactions. The obtained major new results are the following: (i) The incorporation of acemannan and the polyelectrolyte shell significantly influenced the internal structure and population heterogeneity of the cubosomes, with pH playing a crucial modulatory role; (ii) Interactions with mucins (submaxillary and gastric) were pH-dependent, showing increased structural heterogeneity and interaction at pH 7.4, consistent with the known conformational changes of mucin. At pH 2.5, the interactions were minimal; (iii) Pepsin induced greater structural heterogeneity in PS-cubosomes at pH 7.4 due to its pH-dependent conformational changes and increased hydrophobicity, while minimal structural changes were observed at pH 2.5; (iv) Bile salts were potent disruptors of cubosome structure, inducing lamellar phases at pH 2.5 in some acemannan-loaded formulations and complete disassembly of all cubosome types at pH 7.4; (v) Pancreatin induced a phase transition from cubic to H_II_ phase in PS-cubosomes loaded with 30% acemannan at pH 7.4 over time, a process confirmed by RT-SAXS and Cryo-TEM. This effect was dependent on both high acemannan concentration and alkaline pH, suggesting a synergistic role of acemannan in modulating enzyme access or activity. At pH 2.5, pancreatin had minimal effect. (vi) A simulated duodenum mixture containing pepsin, pancreatin, and bile salts led to lamellar phase formation at pH 2.5 for acemannan-loaded systems and complete disassembly at pH 7.4, with bile salts appearing to be the dominant factor for disassembly. These *in situ* results highlighted the complex interplay between the cubosome formulation (lipid, polyelectrolyte shell, and bioactive loading), the surrounding pH, and specific digestive biomolecules. They emphasize the need to consider the full complexity of the GI environment for rationally engineering advanced lipid/biopolymer nanocarriers for effective oral drug delivery with predictable stability and release profiles.

## 5 Materials and Methods

### 5.1 Sample Preparation

The lipid monoolein (1-oleoyl-rac-glycerol, 99%) and copolymer Pluronic F127, both in powder form (Sigma-Aldrich), were dissolved in chloroform at 200 and 100 mg/mL, respectively, to create stock solutions. Different buffers were prepared: pH 2.5 (Glycine-HCl Buffer), pH 4.5 (Acetate Buffer), and pH 7.4 (Phosphate Buffer) to probe the different conditions of interaction in the body relevant to oral treatment. The components were dissolved in separate recipes. Chitosan-N-arginine (CH-arg) (135 kDa), previously synthesized, purified, and characterized,^56^ was dissolved at a concentration of 10 mg/mL in Acetate Buffer pH 4.5. Alginate (Alg) (200 kDa, Sigma-Aldrich) was dissolved at 10 mg/mL in Acetate Buffer pH 4.5 with overnight stirring. Acemannan (BiAloe powder extracted from certified organic *Aloe vera*, provided by Lorand Laboratories, Houston, TX, USA) with MW ∼ 17 kDa, was prepared at 10%, 20%, and 30% of monoolein weight (w/w) and dissolved in MiliQ water.

The stomach proteins were prepared separately in buffers at pH 2.5 and 7.4. Pepsin from porcine gastric mucosa (lyophilized powder, ≥ 2,500 units/mg, Sigma-Aldrich) was prepared at 7 mg/mL. Pancreatin from porcine pancreas (lyophilized powder, Sigma-Aldrich) was prepared at 7 mg/mL. Bile salts (lyophilized powder, Sigma-Aldrich) were prepared at 7 mg/mL. Mucin from bovine submaxillary glands (Type 1-S, ∼ 1,600–2,900 kDa, Sigma-Aldrich) was prepared at 4 mg/mL. Mucin from porcine stomach (Type III, bound sialic acid 0.5–1.5%, partially purified powder, ∼ 640 kDa, Sigma-Aldrich) was prepared at 4 mg/mL. A duodenum mixture of pepsin, pancreatin, and bile salts was prepared with each component at 7 mg/mL, dissolved in buffer pH 2.5 or 7.4.

### 5.2 Small-Angle X-ray Scattering

Small-angle X-ray scattering (SAXS) experiments were conducted at the European Synchrotron Radiation Facility (ESRF) in Grenoble, France, using a SAXS setup equipped with an Eiger X 4 M detector. Each sample was introduced into a flow-through cell and measured at room temperature.

The scattering vector modulus is defined as

q=4πsinθ/λ

where 2θ is the scattering angle and λ is the X-ray wavelength (0.1 Å). The measurements were performed at an energy of 12.3984 keV, with a sample-to-detector distance of 2 m.

For cubic symmetry determinations, the relationship between the lattice parameter a_Q_ and the scattering vector q­(*hkl*) of the diffraction peaks is given by

$$q(\mathrm{hkl})=(2\pi /a_{Q})\times \surd (h^{2}+k^{2}+l^{2})$$

where (hkl) are the Miller indices of the Bragg reflections associated with a given cubic symmetry. By plotting q versus √(h^2^ + k^2^ + l^2^)­ $\sqrt{h^{2}k^{2}l^{2}}$ , the slope of the linear fit to the data corresponds to 2π/a_Q_, allowing calculation of the lattice parameter (a_Q_).

For the inverse hexagonal determinations, the lattice parameter was determined from the scattering vector of the first diffraction peak (q_1_), using the relation:

$$a_{Q}=4\pi /(\surd 3\times q1)$$

This equation derives from the characteristic geometry of the inverse hexagonal phase, in which the diffraction planes correspond to the intercylindrical distances of an ordered two-dimensional lattice.

### 5.3 Cryogenic Transmission Electron Microscopy

Cryogenic transmission electron microscopy (cryo-TEM) was carried out at the Brazilian Nanotechnology National Laboratory (LNNano, CNPEM, Campinas, Brazil). Sample preparation was performed in a controlled environment using an automated vitrification system (Vitrobot Mark IV, FEI, Netherlands). Copper grids (300-mesh, TED Pella, Redding, USA) coated with lacey carbon film were first subjected to glow discharge treatment using an easiGlow system (Pelco) at – 15 mA for 10 s to improve sample adherence and wettability. Subsequently, a 3 μL aliquot of the sample was applied to the grid surface. Excess liquid was removed by automatic blotting using a negative blotting force, after which the grid was rapidly vitrified by plunging into liquid ethane cooled in a liquid nitrogen environment. The vitrified grids were then transferred into cryogenic grid boxes under liquid nitrogen and imaged using a JEOL JEM-2100 transmission electron microscope operated at 200 kV. Data acquisition was performed under low-dose imaging conditions, with the defocus set between 2 and 4 μm to enhance the contrast of internal nanostructures. Micrographs were captured using a CMOS F-416 camera and processed with the EMMENU software (version 4.0.9.52, TVIPS, Germany). Image analysis, including structural evaluation and digital processing, was conducted using ImageJ software, with Fast Fourier Transform (FFT) techniques applied to assess the internal organization of the nanoparticles.

### 5.4 Pending Drop Tensiometry

The surface tension analysis was performed using a KRÜSS Drop Shape Analyzer, model KRÜSS DSA100 (KRÜSS GmbH, Germany), to measure the surface and interfacial tensions at different acemannan concentrations: 0.1 mg/mL, 1 mg/mL, 2 mg/mL, 5 mg/mL, 10 mg/mL, 20 mg/mL, 30 mg/mL, 40 mg/mL, 50 mg/mL, 60 mg/mL, 80 mg/mL, 100 mg/mL, and a mixture containing 7 and 0.7 mg/mL of acemannan in a simulated duodenum solution. The surface tension was measured using the pendant drop technique with a glass syringe needle (diameter: 1.86 mm) in a buffer solution.

## Supplementary Material



## Data Availability

The data that support the findings of this study are available from the corresponding authors upon reasonable request.

## Notes

Additional experimental details and results for surface tension analysis (PDF)

## Notes

All authors approved the final manuscript for submission.
