# Corrections to “Dynamic Self-Assembly and Stimuli-Responsive Disassembly of Bioactive-Loaded Cubosomes in Biomimetic Media Traced by Real-Time Small-Angle X‑ray Scattering and Cryogenic Transmission Electron Microscopy”

**DOI:** 10.1021/acsami.6c01264

**Published:** 2026-01-28

**Authors:** Rafael R. M. Madrid, Angelina Angelova, Borislav Angelov, Gouranga Manna, Patrick D. Mathews, Omar Mertins

**Correction to Figures:**

The *q*-axis units in Figures 3, 4, 5, 6, 7, 8, 9, and 10 have been labeled as inverse Angstroms. The correct unit for the data presented is inverse nanometers. The numerical values on the axis remain unchanged; only the unit label requires correction.

**Correction to section Materials and Methods:**

The technical details regarding SAXS instrumentation and data acquisition in section “5.2. Small-Angle X-ray Scattering” (Page 69130) are updated with the correct parameters below:

Original Text: “The measurements were performed at an energy of 12.3984 keV, ...”

Corrected Text: “The measurements were performed at an energy of 12.23 keV, ...”

Original text: “... using a SAXS setup equipped with an Eiger X 4 M detector. Each sample was introduced into a flow-through cell and measured at room temperature.”

Corrected text: “... using a SAXS setup equipped with an Eiger 2 × 4 M detector. Each sample was introduced into a flow-through cell and measured at room temperature. To minimize radiation damage, the sample was displaced after each exposure.”

We confirm that these errors are strictly limited to the axis labeling and the description of the experimental setup. They do not affect the interpretation of the scattering profiles, the results, or the overall conclusions of the paper.

