# Supplementary material for: Dynamic Self-Assembly and Stimuli-Responsive Disassembly of Bioactive-Loaded Cubosomes in Biomimetic Media Traced by Real-Time Small-Angle X‑ray Scattering and Cryogenic Transmission Electron Microscopy
Source: ACS Appl Mater Interfaces. 2025 Dec 12;17(51):69118–33. doi: 10.1021/acsami.5c18735 (PMC12754745; doi:10.1021/acsami.5c18735)
Supplement: Supplementary file 1 [file am5c18735_si_001.pdf]

Supporting Information

Dynamic Self-Assembly and Stimuli-Responsive Disassembly of Bioactive-Loaded Cubosomes in Biomimetic Media Traced by Real-Time SAXS and Cryo-TEM

Rafael R.M. Madrid,[a] Angelina Angelova,[b]* Borislav Angelov,[c]
Gouranga Manna,[d] Patrick D. Mathews,[e] and Omar Mertins[a]*

[a]Laboratory of NanoBioMaterials - LNBM, Department of Biophysics, Paulista Medical School, Federal University of São Paulo, 04023-062 São Paulo, Brazil
[b]Institut Galien Paris-Saclay, CNRS, Université Paris-Saclay, 91400 Orsay, France
[c]Department of Structural Dynamics, Extreme Light Infrastructure ERIC, 18221 Dolni Brezany, Czech Republic
[d]ESRF, The European Synchrotron, 71 Avenue des Martyrs, 38000 Grenoble, France
[e]Institute of Bioscience, São Paulo State University, 18618-689 Botucatu, Brazil

*E-mail: mertins@unifesp.br (O.M.); angelina.angelova@universite-paris-saclay.fr (A.A.)

Method

The surface tension analysis was performed using a KRÜSS Drop Shape Analyzer, model KRÜSS DSA100 (KRÜSS GmbH, Germany), to measure the surface and interfacial tensions at different acemannan concentrations: 0.1 mg/mL, 1 mg/mL, 2 mg/mL, 5 mg/mL, 10 mg/mL, 20 mg/mL, 30 mg/mL, 40 mg/mL, 50 mg/mL, 60 mg/mL, 80 mg/mL, 100 mg/mL, and a mixture containing 7 mg/mL and 0.7 mg/mL of acemannan in a duodenum solution. The surface tension was measured using the pendant drop technique with a glass syringe needle (diameter: 1.86 mm) in a buffer solution.

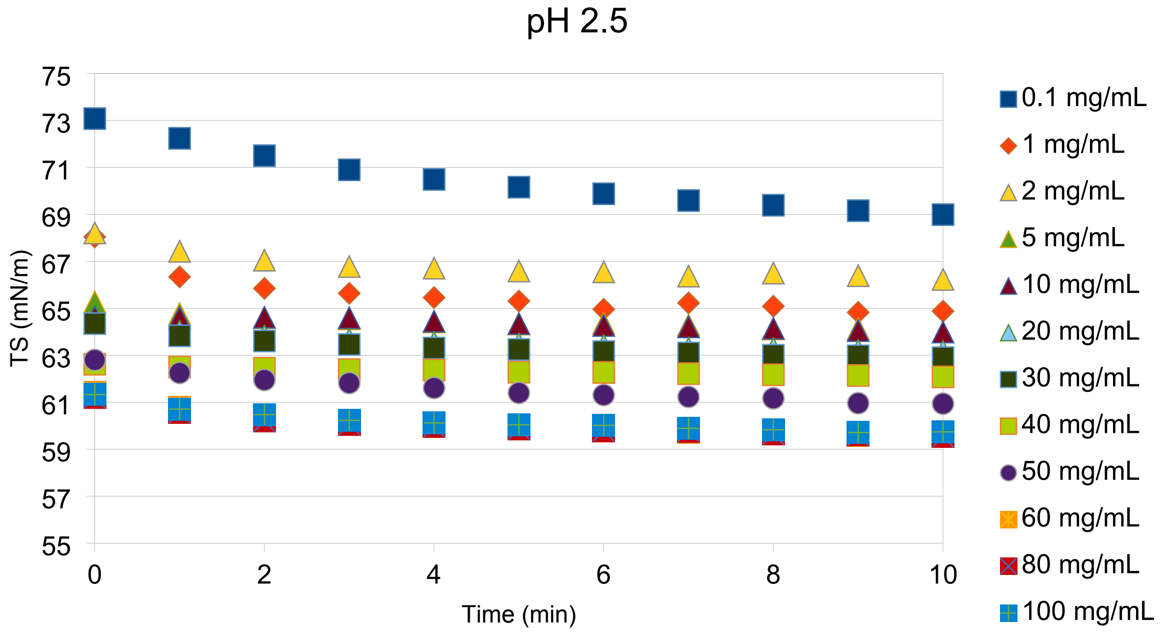

Figure S1. Surface tension dynamics over time under different concentrations of acemannan in buffer at pH 2.5. An increase in acemannan concentration leads to a reduction in the surface tension of the solution, indicating its ability to interact with the interface and form hydrophobic aggregates. This behavior is likely driven by interactions between its polymer chains, promoted by the presence of acetylated regions.

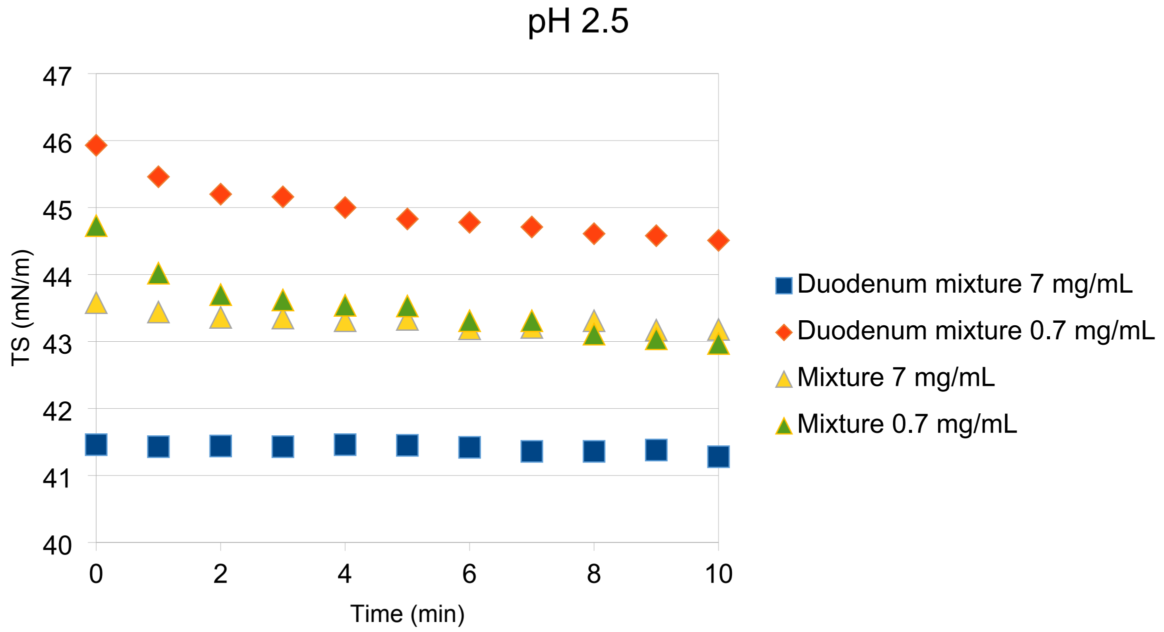

Figure S2. Surface tension dynamics of two different concentrations of duodenum solution (0.7 mg/mL and 7 mg/mL) before and after interaction with acemannan (20 mg/mL) in buffer at pH 2.5. It can be observed that the higher concentration of the duodenum solution exhibits increased hydrophobic properties after interacting with acemannan. The surface tension rises due to the interaction with acemannan, which acts as an emulsifying agent in the solution.

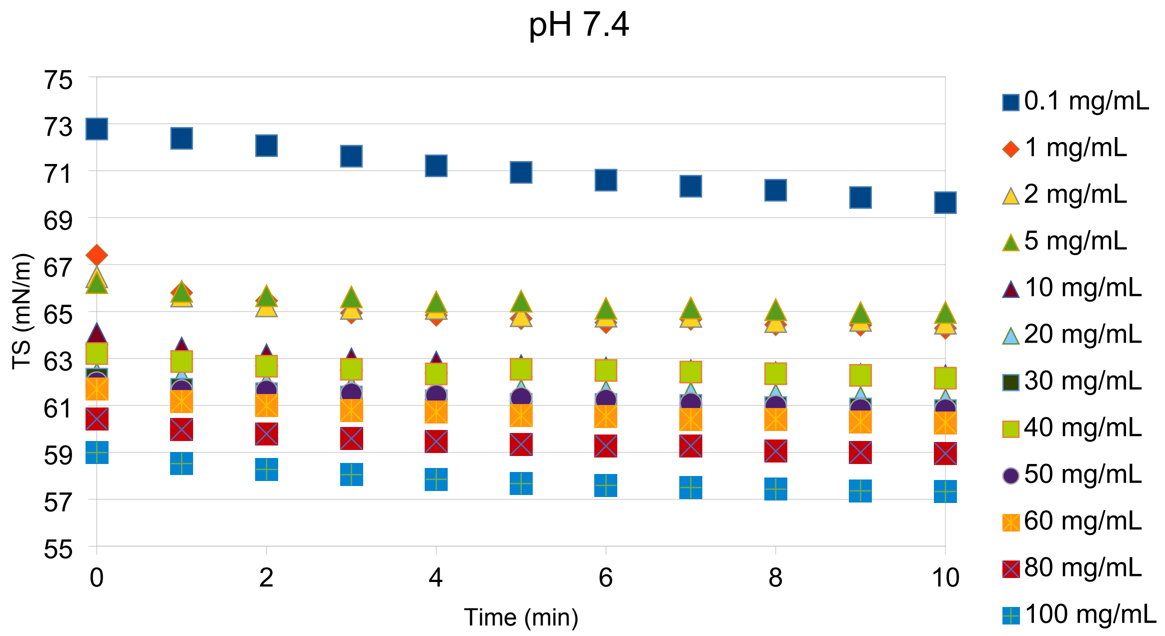

Figure S3. Surface tension dynamics over time under different concentrations of acemannan in buffer at pH 7.4.

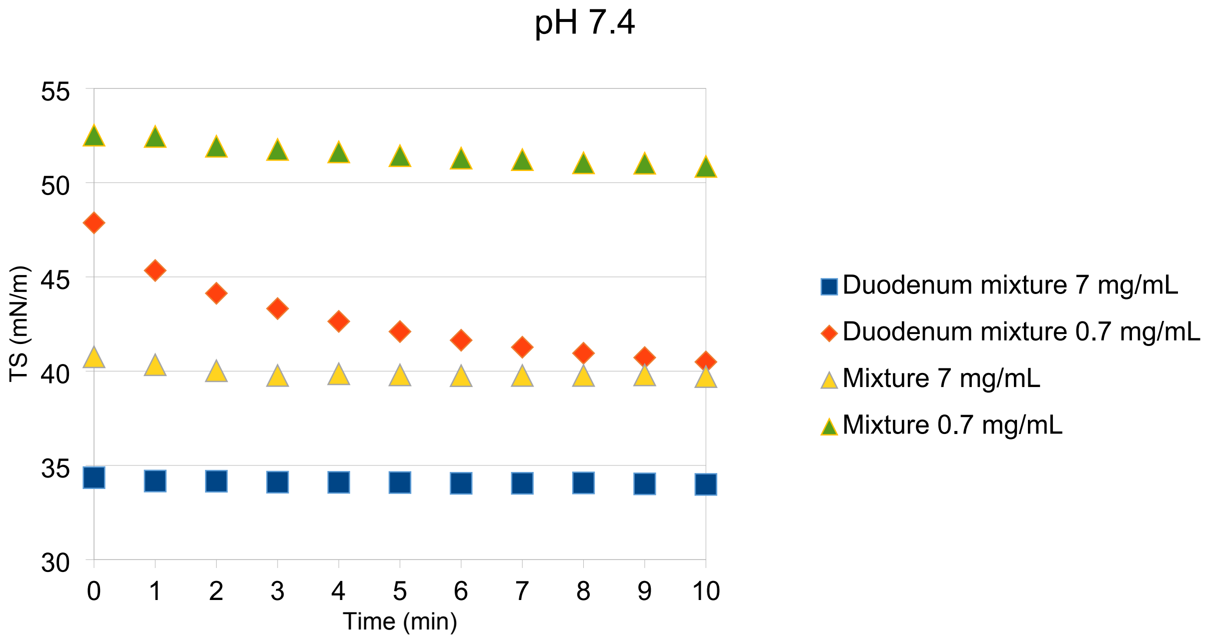

Figure S4. Surface tension dynamics of duodenum solution (0.7 mg/mL and 7 mg/mL) before and after interaction with acemannan (20 mg/mL) in buffer at pH 7.4. After interaction with acemannan (Mixture) at pH 7.4, the system exhibits increased wetting properties, as evidenced by a rise in surface tension of approximately ~6 mN/m at both concentrations. This indicates that acemannan displays enhanced emulsifying capacity under neutral pH conditions.
